# Ursolic Acid Induces Multifaceted Defense Responses Against Postharvest Blue Mold Rot in Apple Fruit

**DOI:** 10.3390/foods14050761

**Published:** 2025-02-23

**Authors:** Chang Shu, Wenxiao Jiao, Kuanbo Cui, Jiankang Cao, Weibo Jiang

**Affiliations:** 1 College of Food Science and Nutritional Engineering, China Agricultural University, Beijing 100083, China; sinosc@126.com (C.S.); cjk@cau.edu.cn (J.C.); 2 College of Food Science and Engineering, Qilu University of Technology, Jinan 250353, China; jiaowx9857@qlu.edu.cn; 3 Agricultural Mechanization Institute, Xinjiang Academy of Agricultural Sciences, Urumqi 830091, China; widewave@126.com

**Keywords:** induced resistance, postharvest decay, ursolic acid, reactive oxygen species metabolism, blue mold rot

## Abstract

The disease resistance and defense mechanisms induced by ursolic acid (UA) in apple fruit were studied in this paper. UA was directly mixed with potato dextrose agar and broth media to assay its antifungal activity in vitro. The results showed that UA exerted inherent antifungal activity and directly inhibited the in vitro growth and spore germination of *Penicillium expansum*. Its half-maximal inhibitory concentration for hyphal growth was 175.6 mg L^−1^. Apple fruit were immersed in UA solution, followed by inoculation with *P. expansum*, to measure their disease response. The results demonstrated that UA induced significant disease resistance in apple fruit and that its mechanisms are multifaceted and associated with defensive and antioxidative enzymes and the phenylpropanoid pathway. Chitinase, β-1,3-glucanase, peroxidase, and polyphenol oxidase were activated and maintained at relatively high levels. The activities of enzymes and their metabolites in the phenylpropanoid pathway, including phenylalanine ammonia-lyase, cinnamate-4-hydroxylase, and 4-coumarate coenzyme A ligase were significantly increased; accordingly, total phenolics, flavonoid, and lignin contents were significantly increased. The activities of superoxide dismutase, ascorbate peroxidase, and glutathione reductase were enhanced upon UA treatment, while catalase activity was suppressed, which regulates hydrogen peroxide accumulation to defend against pathogens. These results suggest that UA induces defense responses against postharvest blue mold rot in apple fruit and that it may be a promising elicitor to induce fruit disease resistance to control postharvest decay.

## 1. Introduction

Apples are one of the most widely consumed fruits due to their appealing flavor and high nutritional value, and they are widely consumed fresh or processed. Despite modern postharvest storage and handling technologies, apple fruit severely suffer from fungal diseases, causing considerable postharvest losses in production [1]. Serval pathogenic fungi can infect apple fruit, for instance, *Botrytis cinerea* (gray mold), *Penicillium* sp. (blue mold)*, Alternaria* (black spot rot), *Colletotrichum* sp. (bitter rot), and *Monilinia* spp. (brown rot) [2,3]. These pathogens not only severely affect the appearance and edible quality, but also cause fruit to lose their commercial value [4]. Under some conditions, *Penicillium* species are capable of generating patulin, a highly detrimental mycotoxin for humans; thus, food safety authorities have focused on this species [5]. Nowadays, fungicidal chemicals are the primary strategy to control blue mold rot. However, sustained application of chemical fungicides will accelerate fungicide resistance, which will lead to toxicological risks and environmental pollution. Consequently, it is necessary to explore novel, non-toxic, low-cost, and effective alternative strategies to reduce postharvest decay [6].

Inducing the host’s inherent disease resistance, i.e., induced resistance, offers an efficient alternative approach to control postharvest losses. The disease responses induced by different elicitors offer the host an effective, endurable, and reliable resistance against a broad spectrum of pathogens [7]. More importantly, this disease resistance remains effective throughout the host’s shelf life, making it a cornerstone of integrated disease management strategies [8]. A range of elicitors have been continuously developed and applied over the last 25 years; typical chemical elicitors including salicylic acid (SA) [9], methyl jasmonate [10], acibenzolar-S-methyl [11], benzothiadiazole (BTH) [12], and oxalic acid [13] play important roles in the industry. Novel elicitors such as ε-poly-l-lysine [1], sodium nitroprusside [2], l-glutamate [14,15], and cell wall components [16,17] have been demonstrated to be effective in inducing disease resistance to reduce postharvest decay. There are two typical modes of induced resistance: systemic acquired resistance (SAR), which is triggered by localized pathogen infections, is dependent on the salicylic acid (SA) signaling pathway and is accompanied by the accumulation of pathogenesis-related (PR) proteins, and systemic resistance (ISR), which is predominantly activated by beneficial rhizosphere microorganisms that enhance broad-spectrum resistance such as cell wall reinforcement through jasmonic acid and ethylene signals [18]. Both achieve long-term protection of distal tissues through the priming effect. In postharvest studies, a series of complex biochemical changes are involved in the establishment and function of induced resistance, including the accumulation of PR proteins, activation of antioxidant systems, and modulation of phenolic metabolism and ripening/senescence processes [7,8,19]. The development and application of new elicitors and the use of various new tools to comprehensively monitor the physiological processes of different produce varieties are important areas of research on induced resistance.

Ursolic acid (UA) is a triterpenoid compound that can be isolated from diverse plant sources, including their stem bark, leaves, and fruit peel. Apart from its widespread human-health-related bioactivities [20], as a key component of fruit cuticular wax, it plays a crucial protective role due to its various biological activities that enhance fruit quality while boosting both stress tolerance and disease resistance [21,22]. Studies have also shown that cuticular wax components may serve as signaling molecules, modulating plant defense mechanisms [23]. As an important component of fruits’ cuticular wax, ursolic acid forms a physical barrier to protect the fruit from external damage and thus maintains its postharvest quality. Ursolic acid is an important component of blueberry cuticular wax [22]. After the wax is artificially removed, the postharvest quality deteriorates rapidly and the decay rate increases [24,25]. Ursolic acid also exhibits inherent antifungal activity, inhibiting *A. alternata* [26], *B. Cinerea* [27], and *Colletotrichum gloeosporioides* [28] in vitro without phytotoxicity [29,30]. Although the mechanism by which ursolic acid directly enhances disease and stress resistance in fruits is not fully understood, extensive research has shown that it plays multiple roles in plant defense mechanisms. UA reduces salt stress-induced oxidative stress in rice via the regulation of nitric oxide metabolism and oxidative defenses [31]. Exogenous UA treatment induces defense responses in postharvest blueberries, significantly activating defense enzymes such as POD, PPO, GLU, CHI, and PAL [32]. Potassium phosphate enhances walnuts’ resistance to anthracnose by inducing ursolic accumulation [28]. UA has also been reported to specifically bind to some enzymes in apples and improve the edible quality of fresh-cut apples by promoting the scavenging of ROS and reducing the microbial population [33]. To date, only one study has investigated UA-induced resistance in postharvest blueberries [32], and the mechanism by which UA elicits resistance in other fruit, as well as the systematic defense responses of the hosts, has not been elucidated.

Our previous research indicated that UA exerts antifungal activity toward *A. alternata* and induces defense enzyme activities in postharvest apples [26], suggesting UA may be a potential elicitor to control postharvest decay. This study investigated disease-resistance-related enzymes, phenylpropane metabolism, and ROS scavenging to reveal the multifaceted mechanisms of ursolic acid-induced disease resistance responses in postharvest apples. This research provides potential mechanisms for how UA induces defense responses in apple fruit and reduces postharvest decay.

## 2. Materials and Methods

### 2.1. Fruit and Microbial Material

Apples (Fuji) were harvested at a commercial orchard in Beijing, China, and delivered by truck to the laboratory within 2 h of harvesting. The fruit were of similar size, weight, and appearance, with no pathogen infections or physical injuries. Sodium hypochlorite solution 1% (*v*/*v*) was utilized for surface disinfection; afterward, the fruit were rinsed and then dried naturally at room temperature.

*Penicillium expansum* was cultivated at 28 ± 0.5 °C for ten days using the following method: 6–8 mL distilled water was pipetted into a well-sporulating plate and then filtered through four layers of cheesecloth to prepare a spore suspension. The concentration was adjusted to 1 × 10^6^ spores mL^−1^ and the suspension was kept at 4 °C in the dark.

### 2.2. Antifungal Activity Measurement

Spore germination and colony inhibition were measured using a previous protocol [26] with some modifications. For spore germination, the conidial suspension was mixed with PDB media containing UA at concentrations of 25 to 150 mg L^−1^. Spore germination was microscopically assayed after 6, 8, 10, and 12 h at 28 °C. Spores were judged to be germinated once their tube length reached the spore diameter. More than 200 spores from 5 random view fields were observed, and each test was performed in 3 replicates; the results are presented as a percentage.

To assess colony growth, 2 μL of spore suspension was gently dipped in potato dextrose agar (PDA) media containing UA at concentrations of 25 to 150 mg L^−1^. The colony diameter was measured after incubation at 28 °C for 7 days. Inhibition was assayed using Formula (1):

(1) $$Inhibition\;rate\;\left(\%\right)=\frac{\left(\emptyset 1-\emptyset 2)-(\emptyset 3-\emptyset 2\right)}{\emptyset 1-\emptyset 2}\times 100\%$$

where ∅1 represents the colony diameter of the control, ∅2 denotes the diameter of the dipped spore suspension (un-germinated colonies are considered to have a diameter of 5 mm for a 2 μL spore suspension), and ∅3 is the colony diameter of UA groups [34]. The IC_50_ value was determined using the logarithm model.

Mycelium cultivation was conducted according to a previous study [35]. The spore suspension was combined with PDB media, and UA was added to the mixture to obtain mixtures with concentrations of 25 to 150 mg L^−1^. The tubes were incubated at 28 °C, mycelial pellets were collected every 24 h, and they were eventually dried at 60 °C until a constant weight was achieved.

### 2.3. UA Concentration on the Induced Resistance

UA concentrations were chosen with reference to our preliminary experiments and previous study. Apples were vacuum infiltrated with UA solutions (0, 25, 50, 100, and 150 mg L^−1^) using the following procedure: the pressure was reduced to −0.02 MPa using a vacuum pump over two minutes and stabilized at −0.02 MPa for two minutes. Finally, the chamber was gradually restored to atmospheric pressure over another 2 min [26]. The treated fruit were maintained at ambient temperature and induced for 24 h. The fruit were pierced by a sterile borer to create 4 mm deep and 2 mm wide wounds on the two opposite equatorial sides of each fruit, and then they were inoculated with the suspension (30 μL) and maintained at 25 °C and 80% relative humidity (RH). The fruit were wrapped in polyethylene bags (with an average pore size of <0.04 mm). After eight days, the lesion size was recorded using the decussation method with a vernier caliper; each lesion was measured twice in a perpendicular manner.

### 2.4. Induction Time Interval for the Induced Resistance

To optimize the induction timing and understand the resistance mechanisms for enhancing disease control strategies, the induction time interval was examined. Fruit were vacuum infiltrated with 100 mg L^−1^ UA as described in Section 2.3. After inducing for 12, 24, 36, and 48 h, the fruit were pierced and inoculated with the spore suspension and then maintained at 25 °C and RH = 80%. The disease size was measured on the 8th day post-inoculation.

### 2.5. UA-Induced Resistance Against Blue Mold Rot in Harvested Apple Fruit

Apples were vacuum infiltrated with 100 mg L^−1^ UA, pierced, and inoculated with spore suspension after 24 h of induction, and then stored in plastic containers for ten days. The lesion diameter and disease incidence were recorded during storage [36]. The tissue around the wounds was excised between 0.5 and 1.5 cm under the epidermis of ten fruit at each time point. This tissue was cut into cubes (3–5 mm^3^) and frozen at −80 °C for phytochemical and enzyme activity determination.

### 2.6. Total Phenolics, Flavonoid, and Lignin Contents

Secondary metabolites were determined using a previously described protocol [2]. The Folin–Ciocalteu method was employed to measure the total phenolics content. The total phenolics and flavonoid contents are expressed in g kg^−1^, while the lignin content is represented as a percentage.

### 2.7. Hydrogen Peroxide Content

H_2_O_2_ levels were determined using a previous method [37]. Frozen tissue was ground with cold acetone, and sample extraction was monitored using the colorimetric method. The absorbance was measured at 410 nm using a spectrophotometer, and the results are expressed in μmol kg^−1^ FW.

### 2.8. Measurement of Enzymatic Activities

Defense enzyme activities were measured using a previous method [38]. According to the dinitrosalicylic acid (DNS) method, the absorbance was measured at 540 nm, and the β-1,3-glucanase (GLU) activity was expressed as the amount of glucose released per second per gram fresh weight [39]. For chitinase (CHI), colloidal chitin was the substrate, and the dimethylaminobenzaldehyde (DMAB) method was used to measure the absorbance at 585 nm [40].

A previously described protocol [41] was used to evaluate the activity of peroxidase (POD) and polyphenol oxidase (PPO). The increase in absorbance at 470 nm and 420 nm was monitored, indicating the formation of tetraguaiacol [42] and quinones, respectively. The PPO activity was expressed as the change in absorbance per minute per gram of fresh weight [43].

Superoxide dismutase (SOD) and catalase (CAT) activities were determined using previous methods. The absorbance increase at 560 nm was assayed: one unit of SOD activity is defined as the amount of enzyme required to halve NBT reduction per milligram of protein [42]. For CAT activity, the absorbance was measured at 240 nm, and the activity was expressed as the amount of H_2_O_2_ decomposed per minute per gram of fresh weight [44].

Ascorbate peroxidase (APX) activity was determined using a previous protocol [45]. The absorbance was monitored at 290 nm, indicating ascorbate oxidation, and the enzyme activity was calculated as μmol ascorbate oxidized per minute per milligram of protein [46].

Glutathione reductase (GR) was calculated based on the extinction coefficient of NADPH; the result is expressed as μmol NADPH oxidized per minute per gram of fresh weight [47].

Key enzymes involved in the phenylpropanoid pathway were measured using a spectrophotometric method. The phenylalanine ammonia-lyase (PAL) level was determined by monitoring the absorbance at 290 nm and calculating the activity using the extinction coefficient of trans-cinnamic acid, and was expressed in per milligram of protein [38]. Cinnamate-4-hydroxylase (C4H) activity was assessed by measuring the conversion of trans-cinnamic acid to *p*-coumaric acid at 290 nm. C4H activity was expressed as the amount of *p*-coumaric acid formed per hour per gram of fresh weight [48]. For 4-Coumarate coenzyme A ligase (4CL), its activity was calculated based on the extinction coefficient of 4-coumaroyl-CoA per hour per gram of fresh weight [49].

The Bradford method was applied to measure protein concentrations in fruit tissue [50].

### 2.9. Data Analyses

Statistical analyses were performed using IBM SPSS Statistics software 22.0 (IBM Corporation, Armonk, NY, USA). All the experiments were performed in triplicate, with the results presented as means accompanied by their standard errors (SEs). To evaluate the differences among experimental groups, a one-way analysis of variance (ANOVA) was performed, followed by post hoc comparisons using Duncan’s multiple range test. Results were considered statistically significant when the probability value (*p*) was less than 0.05. Prior to analysis, data were verified for normality and homogeneity of variance to ensure the appropriateness of parametric testing. Graphical representations of the data were generated using OriginPro 2017 software.

## 3. Results and Discussion

### 3.1. P. expansum Was Inhibited by UA In Vitro

**Figure 1:**
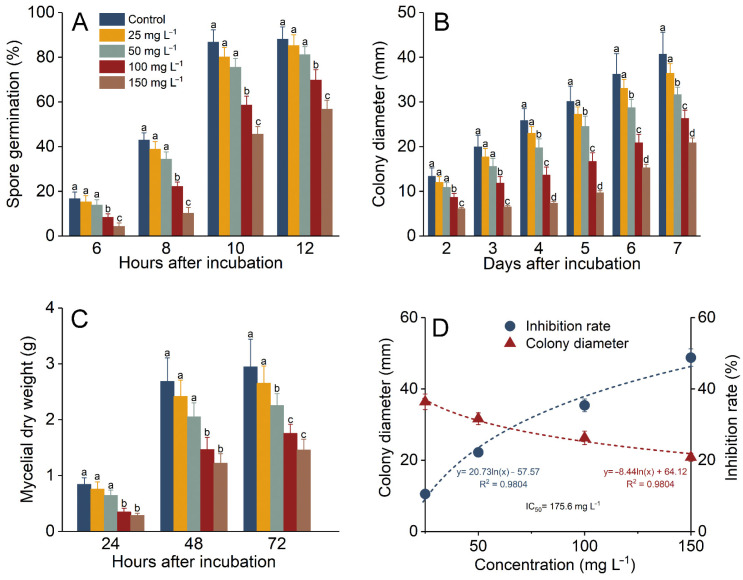
Inhibitory effects of ursolic acid on *P. expansum* growth in vitro. *P. expansum* spore germination (**A**), colony diameter (**B**), and mycelial production (**C**) were significantly reduced by UA when compared to the control. The relationship between the UA concentration and inhibition rate can be described by a logarithmic model (**D**). Results are presented as means ± standard error, with different letters indicating significant differences (*p* < 0.05).

UA exerted antifungal activity in vitro, significantly inhibiting spore germination, colony growth, and mycelial growth, and the inhibition was related to UA levels. In the presence of UA, spore germination was inhibited at the beginning of incubation. After 6 h of incubation, 16.8% of the spores in the control group germinated, while 100 and 150 mg L^−1^ of UA significantly inhibited germination, with inhibition rates of 49.3% and 74.27%, respectively. After 12 h of incubation, 88.2% of the spores in the control group had germinated, while the inhibition rates for spores treated with 100 and 150 mg L^−1^ of UA were 20.74% and 35.5%, respectively (Figure 1A). Similarly, the colony diameter was 22.3% and 48.8% smaller on day 7 than that of control after treatment with 50 and 150 mg L^−1^ UA, respectively (Figure 1B). After 72 h of incubation, the mycelial dry weight was 50.7% lower than that of the control when incubated in 150 mg L^−1^ UA (Figure 1C), inhibited by 40.5% when compared to the control. By calculating the logarithmic model between UA concentration and colony diameter inhibition, the half-maximal inhibitory concentration (IC_50_) of UA was determined to be 175.6 mg L^−1^, with a correlation coefficient of 0.98, indicating a good concentration-inhibition effect (Figure 1D).

UA is a natural secondary product whose direct antimicrobial effect is closely related to its chemical structure [51]. The highly lipophilic molecular skeleton of UA can interact with the plasma membrane of pathogens, disrupting its integrity and functionality and thereby directly exerting antifungal properties [26,27,52]. Previous studies revealed that UA exerts direct antifungal activity against a wide range of pathogenic fungi, including *A. alternata* [26], *Botrytis cinerea* [27], and *Penicillium* species [29]. Its antimicrobial mechanism was also proven to be related to the induction of oxidative stress in pathogen cells [53]. This multifaceted antimicrobial mechanism, coupled with its safe consumption, makes it a useful product to inhibit the proliferation of microorganisms on the surface of ready-to-eat apples [33]. However, compared with UA’s direct antifungal properties, inducing the inherent resistance of the host can offer disease protection at lower elicitor concentrations. This disease resistance is effective, although the hosts do not develop disease resistance immediately, instead going into a state of alert [54]. Once triggered by specific conditions, such as a microbial infection, the host will produce a series of complex defense responses directly involved in this resistance. In addition, this disease resistance is very long-lasting and can continue during the shelf-life of the host [8]. Some studies have even shown that, in plants, induced resistance can be passed on to the next generation [55]. Although some chemical elicitors such as BTH [12] exhibit no significant antifungal activity against pathogens, some exert significant antifungal activity in vitro. *p*-Coumaric acid not only induces defense responses against postharvest infection in jujubes but also significantly inhibits pathogen growth in vitro [40]. The present results corroborate previous findings that ursolic acid (UA) directly inhibits pathogens [26,30], with a similar antifungal efficiency to a previous study [29].

### 3.2. Influence of UA Concentration and Induction Time Interval on the Development of Apple Fruit Decay

**Figure 2:**
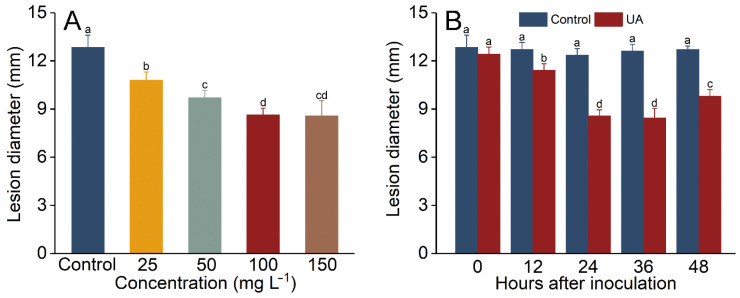
Effects of UA concentration and induction interval on blue mold rot in apples. Impact of UA concentration (25–150 mg L^−1^) on the reduction in blue mold rot caused by *Penicillium expansum* in apple fruit stored at 25 °C for 8 days (**A**). Effect of induction interval (12–36 h) on the reduction in blue mold rot in apple fruit treated with UA (**B**). Distilled water-treated fruit served as the control. Results are presented as means ± standard error, with different letters indicating significant differences (*p* < 0.05).

UA treatment at 25–150 mg L^−1^ could significantly inhibit the lesion diameter in apple fruit (Figure 2A). UA at 100 mg L^−1^ led to the highest inhibition (32.8%) when compared to other concentrations. When the UA concentration was increased to 150 mg L^−1^, the reduction in lesion diameter was not significant when compared to that at 100 mg L^−1^. When the induction interval time was 12 to 36 h, the lesion diameter was significantly suppressed, and the inhibition rate was between 10.1% and 35.4% (Figure 2B). Therefore, the optimal induction time interval was 24 h.

Plants normally require a certain time interval in order to activate a series of defense responses against pathogen infestation, known as the priming effect [56]. A variety of stimuli, including environmental and biological stimuli and elicitors, induce the host to enter a priming phase. A series of molecular and biochemical changes occur in the host during this stage [19], allowing the host to shift the allocation of resources from primary metabolism (for growth and production) to secondary metabolism (for defense), which results in a more intense response once the host is re-infested by the pathogen [8,57]. Previous studies have indicated that the resistance induced by chemical elicitors usually takes 24 h or longer to establish, with the induction time affected by elicitor types and species and temperature [7]. The results of this study are similar to those reported previously [12,38,56], suggesting that UA-induced resistance is highly applicable and similar to current elicitors.

### 3.3. UA-Induced Resistance Offers Protection from Blue Mold Rot in Apples

**Figure 3:**
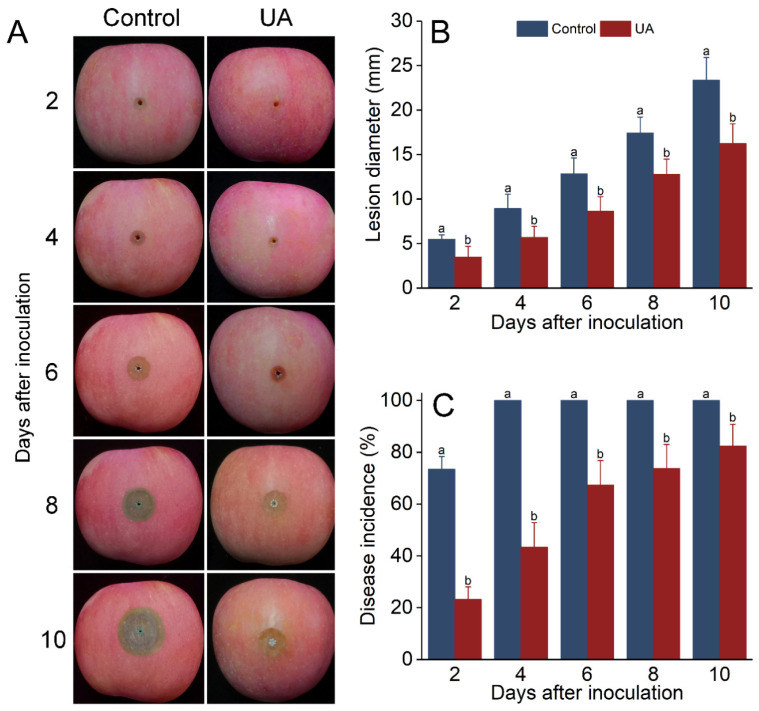
Effects of UA treatment on blue mold rot development in apples. Decay development (**A**), lesion diameter (**B**), and disease incidence (**C**) of apple fruit incubated with *P. expansum* and treated with UA or distilled water (control) during storage at 25 °C for 10 days. Results are presented as means ± standard error, with different letters indicating significant differences (*p* < 0.05).

The lesion diameter and incidence of inoculated fruit gradually increased during storage, while UA significantly inhibited this upward trend (Figure 3A,B). The lesion area and diameter of UA-treated fruit were significantly smaller than those of the control in all cases. The lesion diameter was 30.5% smaller than that of the control after 10 d of storage. Linear regression of the lesion diameter showed that the increase in lesion size in the control group was 2.23 mm per day (R^2^ = 0.993), whereas in the UA group, it was 1.60 mm per day (R^2^ = 0.993). UA also reduced the disease incidence during storage, which was consistently lower than that of the control (Figure 3C). These results indicate that UA induction can significantly inhibit the germination and growth of *P. expansum* in apples and can directly inhibit the germination and infestation activity of spores in the wound site and reduce their morbidity. As for germinated spores, UA induction can significantly inhibit the severity of decay.

The purpose of induced resistance is to delay the onset and development of pathogen infections [8], and our result revealed the potential of UA-induced resistance in postharvest rot control. Inoculating *P. expansum* spores into the wound triggered a rapid and strong response in the wound tissue of the fruit—which was in its priming phase—which is thought to be a result of the increased sensitivity of the plant’s immune system [55,56]. The germination of spores at the wound site was directly inhibited, and the reduced colonization and infectivity were the main reasons for the reduction in incidence rate. A similar phenomenon has also been reported in the resistance induced by chlorogenic acid in harvested peaches [38]. For pathogens that have germinated and infected tissues, the induced resistance response continues to work around the lesions, alleviating the development of the disease [58,59]. *Penicillium* has powdery, aerial spores, increasing its ability to spread and cause large-scale losses [60]. Even if the disease severity can only be partially reduced, the total loss caused by the diseased fruit as a source of contamination could be avoided. Malho et al. [29] and Shaik et al. [30] studied UA’s ability to inhibit citrus blue mold and sorghum fungal diseases, respectively, and reported that its mechanism is related to the accumulation of SA. However, there are differences in induced resistance among plants, mutant fruits, and non-mutant fruits, and research on the UA-induced resistance response of apples is still very limited.

### 3.4. Effects of UA-Induced Resistance on Defense-Related Enzymes in Harvested Apples

**Figure 4:**
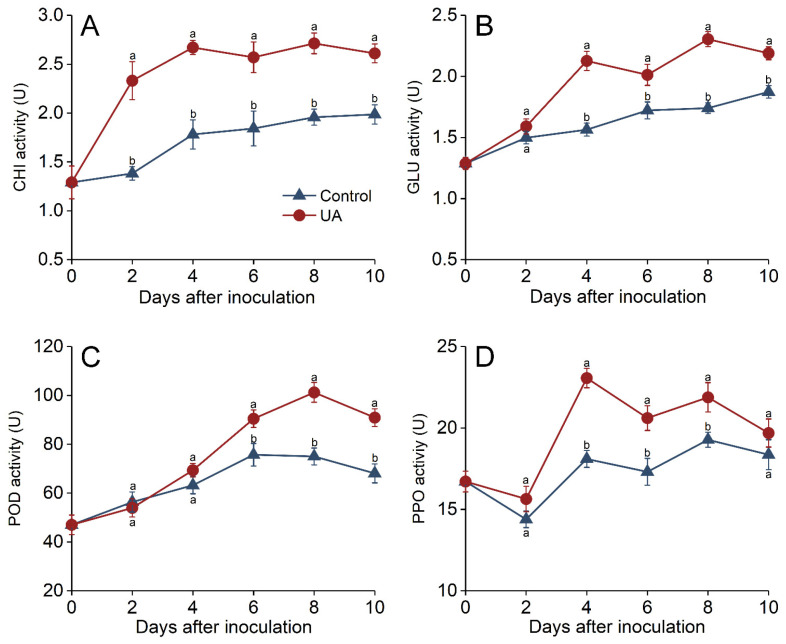
Effects of UA treatment on the activities of defense-related enzymes in apples. Chitinase (**A**), β-1,3-glucanase (**B**), peroxidase (**C**), and polyphenol oxidase (**D**) activities in apple fruit inoculated with *P. expansum* and treated with UA or distilled water (control) during storage at 25 °C for 10 days. Results are presented as means ± standard error, with different letters indicating significant differences (*p* < 0.05).

The CHI activity was significantly enhanced by UA treatment, peaking on day 4, and was 50% higher than that of the control. It was then maintained at higher levels than the control throughout incubation (Figure 4A). The GLU activity also increased in response to UA treatment and was maintained at a remarkably higher level than that of the control (Figure 4B). The GLU activity peaked on days 4 and 8, at 35.9% and 35.3% higher than that of the control, respectively. The POD activity increased continuously and then subsequently decreased (Figure 4C); it was significantly higher than that of the control after 6 to 10 days of incubation. The PPO activity peaked on day 4 and then decreased afterward; it was significantly higher than that of the control 4 to 8 days after incubation, at 12.9% to 27.1% higher than the control (Figure 4D). These results suggest that UA treatment induced an increase in the activities of defense-related enzymes.

PR proteins play an important role in induced resistance in plants, functioning in both SAR and ISR. PR proteins enhance disease resistance by directly suppressing pathogen growth or reinforcing plant cell wall structures [61]. SAR is explicitly dependent on the SA signaling pathway and is characterized by a substantial accumulation of PR proteins. Although ISR is not characterized by this substantial PR protein accumulation, PR protein expression may still be upregulated under certain conditions. In this study, UA treatment significantly induced the activity of PR enzymes. CHI inhibits fungal growth by degrading chitin in fungal cell walls, while GLU enhances resistance by hydrolyzing β-1,3-glucans in fungal cell walls. The significant activation of these enzyme activities facilitates direct suppression of pathogen proliferation at infection sites [1,38,62]. Cell wall lignification significantly triggers disease resistance by enhancing mechanical strength, forming chemical barriers, restricting nutrient acquisition by pathogens, and synergizing with other defense mechanisms [39,56]. This process is critical in limiting pathogen spread and amplifying localized defense responses. PPO contributes to lignification by catalyzing the oxidation of phenolic compounds, promoting lignin precursor polymerization. Similarly, POD strengthens cell walls through lignin biosynthesis, thereby impeding pathogen invasion and colonization [63]. Previous studies have demonstrated that elicitors also induce these defensive enzyme activities. Methyl jasmonate and BTH treatment elevated CHI and GLU activities in mango and jujube, respectively [64,65], while alginate oligosaccharides increased PPO and POD activities in kiwi and pear [66,67]. These findings align with our results, further supporting the hypothesis that ursolic acid enhances apple disease resistance via PR protein activation.

### 3.5. Phenylpropanoid Metabolism Regulation Induced by UA

**Figure 5:**
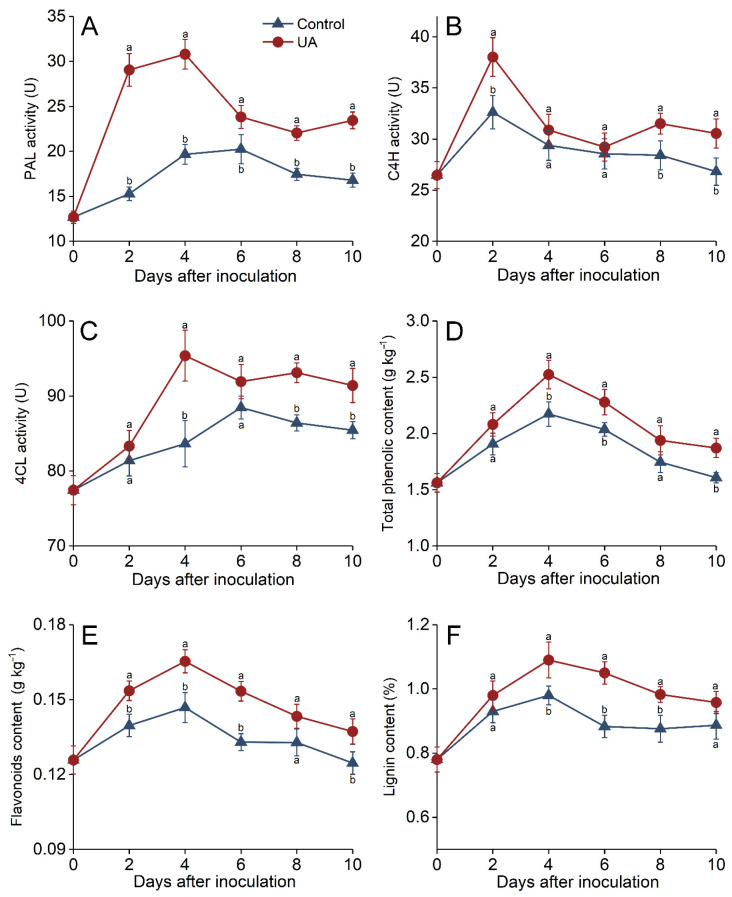
Effects of UA treatment on phenylpropanoid metabolism and secondary metabolite accumulation in apples. Phenylalanine ammonia-lyase (**A**), cinnamate 4-hydroxylase (**B**), and 4-coumarate CoA ligase (**C**) activities, and the content of total phenolics (**D**), flavonoids (**E**), and lignin (**F**) in apple fruit inoculated with *P. expansum* and treated with UA or distilled water (control) during storage at 25 °C for 10 days. Results are presented as means ± standard errors, with different letters indicating significant differences (*p* < 0.05).

Upon UA treatment, PAL activity increased significantly within the first 4 days after inoculation, peaking on day 4 before gradually declining, while its activity in the control fruit exhibited a slow upward trend. On day 4, the PAL activity in UA-treated fruit was 57.1% higher than that of the control. The control group reached its peak 6 days after inoculation; its activity was 15.03% lower than that of the UA group at this time point (Figure 5A). C4H activity increased rapidly and peaked 2 d after inoculation, with the UA group peak being 16.6% higher compared to the control. Furthermore, the C4H activity in the UA group remained significantly elevated over that of the control from day 8 to 10 (Figure 5B). Similarly, the 4CL activity in UA-treated fruit increased rapidly, with a peak on day 4, which was 14.1% higher than that of the control. UA-treated fruit also had a significantly higher 4CL activity than the control from day 8 to 10 (Figure 5C). Concurrently, UA treatment enhanced phenylpropanoid metabolite accumulation in lesion-adjacent tissues (Figure 5D–F). The total phenolics, flavonoid, and lignin levels in UA-treated fruit rose rapidly within the first 4 days; their peak levels were 19%, 14.3%, and 11.2% higher than those of the control, respectively. Although all metabolites gradually declined after day 6, UA-treated groups consistently had significantly higher levels than the control from day 6 to 10.

Phenylpropane metabolism accounts for a significant portion of plant disease resistance, regulating defense compounds through coordinated enzymatic cascades. PAL initiates the first step by converting phenylalanine to cinnamic acid, while C4H hydroxylates cinnamic acid to 4-coumarate. 4CL subsequently activates coumarate derivatives for downstream biosynthesis of phenolics, flavonoids, and lignin. Polyphenolics and flavonoids are antimicrobial compounds and ROS scavengers that suppress pathogens and alleviate oxidative damage during infection [17,68]. Lignin polymerization reinforces cell walls to physically impede pathogen invasion and restrict hyphal spread, a process regulated by spatial–temporal deposition of monolignols [69]. In this study, UA induction significantly enhanced the phenylpropanoid metabolism in the tissues surrounding infection sites, as evidenced by upregulated PAL activity and elevated total phenolics, flavonoid, and lignin levels. These results corroborate recent findings that elevated phenylpropane metabolites are correlated with fruit disease resistance [2,40,68]. Furthermore, lignin’s dual role as a physical barrier and a signaling hub for the expression of defense genes underscores its strategic importance [70]. This result highlights phenylpropanoid metabolism as an important contributor to UA-induced resistance.

### 3.6. Effects of UA on Oxidative Stress Regulation in Apple Fruit

**Figure 6:**
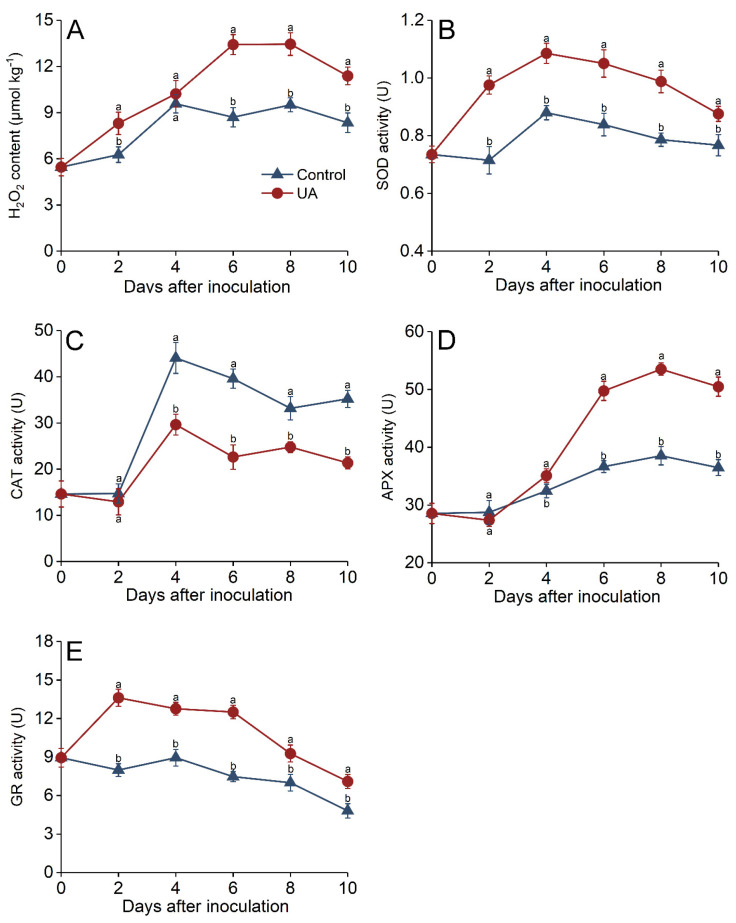
Effects of UA treatment on oxidative burst and antioxidant enzyme activities in apples. Hydrogen peroxide content (**A**) and the activities of superoxide dismutase (**B**), catalase (**C**), ascorbate peroxidase (**D**), and glutathione reductase (**E**) in apple fruit inoculated with *P. expansum* and treated with UA or distilled water (control) during storage at 25 °C for 10 days. Results are presented as means ± standard errors, with different letters indicating significant differences (*p* < 0.05).

H_2_O_2_ levels in UA-treated fruit increased steadily after inoculation and peaked on day 6, subsequently decreasing slightly. They were maintained at significantly higher levels than the control at 6 and 10 days after inoculation, at 1.54- and 1.36-fold higher than the control, respectively (Figure 6A). ROS, including H_2_O_2_, play multifaceted roles in plant disease resistance. As important signaling molecules, they trigger systemic resistance by activating MAPK cascades and NPR1-mediated phosphorylation, thereby upregulating defense genes. Their direct antimicrobial activity arises from the attack of pathogenic cellular components, as well as from participation in quinone formation [71]. H_2_O_2_ also reinforces cell walls via lignin polymerization and oxidative cross-linking of structural proteins. Plants precisely regulate their H_2_O_2_ contents via enzyme machinery, consisting of NADPH oxidase, SOD, and APX/GR catalyzed scavenging, while catalase (CAT) prevents excessive accumulation. Hydrogen peroxide levels and antioxidant enzyme regulatory systems influence host resistance [7]. The observed increase in H_2_O_2_ levels in UA-treated fruit reflects a coordinated regulation of ROS-generating and -scavenging systems. This result is consistent with studies showing that an oxidative burst occurs in fruit during the early phases of plant defense responses [1,39,41].

The SOD activity in the UA group rapidly increased and reached its peak 4 days after inoculation, and was 24.1% higher than the control (Figure 6B). The CAT and APX activities were slightly inhibited 2 days after inoculation and afterward increased with further storage. Four days after inoculation, the UA group exhibited a declined CAT level, which was 32.7% lower than that of the control (Figure 6C). Conversely, the APX activity was enhanced by UA treatment, peaking 8 days after incubation, and was 38.9% higher than the control (Figure 6D). The GR level in the UA group peaked 2 days after inoculation and was maintained at a high level till 6–10 days, while it decreased continuously in the control fruit and was 68.9% lower than the control after 6 days of incubation (Figure 6E).

The initial antioxidant response was dominated by phenolics and glutathione, which are regulated by the ascorbic acid–reduced glutathione (AsA-GSH) cycle and phenylpropanoid pathway, respectively [72]. Our results showed a significant UA-induced elevation in SOD, APX, and GR activities, but a lower CAT activity in apple fruit upon UA treatment. Our results suggest that UA may regulate the antioxidant metabolism and contribute to the accumulation of H_2_O_2_. Meanwhile, the ROS scavenging system was promoted to regulate the cytotoxicity of H_2_O_2_. A similar conclusion has been reported in apples subjected to ε-poly-l-lysine [1] and biocontrol agent [39] elicitors. Several enzymes and compounds that participate in ROS scavenging in plant cells can detoxify excess ROS. Among these, O^2−^ free radicals convert to H_2_O_2_ under SOD catalysis. The formed H_2_O_2_ is scavenged and catalyzed by CAT and APX, which decompose H_2_O_2_ into water and oxygen, or metabolize it in the AsA-GSH cycle, which is dominated by GR [73].

Our previous study found that no phytotoxicity or quality deteriorations were observed in the UA-treated apples [26]. The increasing number of studies on the extraction and synthesis of UA reveals its potential for commercialization and encourages researchers to explore a wider range of applications in the future. It is worth noting that biochemical and transcriptional analyses will be required to evaluate whether treating apple fruit with UA is a novel, effective procedure for reducing postharvest fruit decay and economic losses. Subsequent studies should test the induced resistance of UA in more fruits and against more pathogens and continue to measure fruits’ disease resistance response under more conditions such as different storage temperatures.

## 4. Conclusions

The results demonstrated that ursolic acid effectively enhanced disease resistance in apples and reduced blue mold severity. UA exhibited antifungal activity in vitro, with an IC_50_ against *P. expansum* of 175.6 mg L^−1^. The in vivo results showed that the optimized induction concentration is 100 mg L^−1^ with an induction time interval of 24 h. UA offers fruit a protective effect against blue mold rot, which is attributed to the introduction of multifaceted defense mechanisms. The activities of defense enzymes (CHI, GLU, POD, and PPO) were significantly activated, and the accelerated regulation of bio-pathways (enhanced enzyme activities in the phenylpropanoid pathway, including PAL, C4H, and 4CL) and accumulation of antimicrobial substances (phenolics, flavonoids, and lignin contents) were observed. The regulation of oxidative stress also contributes to disease resistance, as does the significant enhancement in SOD, APX, and GR activities, while CAT activity is suppressed upon UA induction, regulating H_2_O_2_ accumulation to defend against pathogens. These results suggest that UA may serve as a potential elicitor to control and manage postharvest decay in harvested fruit.

## Data Availability

The data presented in this study are available on request from the corresponding author.
